# Commencements

**Published:** 1891-05

**Authors:** 


### COMMENCEMENTS.

### The American College of Dental Surgery, Chicago. Commencement.

The American College of Dental Surgery Chicago, held its commencement exercises at Central Music Hall March 25th, 1891. Rev. S. M. Crissman led in prayer, and L. D. McIntosh, M.D., D.D.S., president of the college, conferred the degree. The Faculty and students were dressed in caps and gowns, and presented a most imposing scene to the large audience that greeted them. The class were:

| NAME. | RESIDENCE. |
|---|---|
| Ernest Phillips Bender | Chicago. |
| Thomas Jefferson Randall | Tenn. |
| William Frackelton | Wisconsin. |
| Sterling Decatur Tuttle, Jr. | Ohio. |
| Joseph Ephraim Derby | Chicago. |
| Henry Williams McIntire | Minn. |
| John Henry Wiede, M.D | Germany. |
| Mrs. Marie Weihe Wiede | Germany. |
| Earl Edwin Murdock | Wisconsin. |
| Joseph Sidney Goodmanson | Minn. |
| Timothy Rogers | Illinois. |
| Mrs. Elia A. Magnusson | Chicago. |
| Richard M. McKey | Chicago. |
| Charles Ellsworth Douglas | Ohio. |
| Aleck Magnus Swan | Chicago. |
| Francis A. Carter | Arkansas. |
| Frank B. Hinchman | Michigan. |
| Charles W. Stanfield | Ontario. |
| Charles E. Ward | Chicago. |
| John Wilmot White | Chicago. |
| Frank Reed Howdle | Iowa. |
| Francis P. Burchell | Iowa. |
| William Frank Mitchell | Illinois. |
| William Charles Bruening | Germany. |
| William Farrell Reber | Chicago. |
| F. A. Valentine Moller | Denmark. |
| Hermann Huelsebusch | Germany. |
| Ernest Alfred Kohler | Chicago. |
| Frederick Bruce Cooker | Chicago. |
| John Franklin Kyler | Illinois. |
| Henry Suesskind | Chicago. |
| Henry Charles Waack | Illinois. |
| George Grant Barlow | Wisconsin. |
| John Frederick Eldred | Illinois. |
| William F. Gilroy | Chicago. |
| William Thomas Jefferson | Chicago. |
| Matthew Kult | Wisconsin. |
| Charles H. McCarty | Chicago. |
| Norris Huston Teal | Wisconsin. |
| James Arthur Garland | Chicago. |
| George Augustus Stevenson | Indiana. |
| H. L. Tilsner | Wisconsin. |
| Peter Ellsworth Walter | Illinois. |
| Frank J. Shea | Chicago. |
| Miss Annie S. Bowers | Chicago. |
| Joy L. Frink | Iowa. |
| W. T. Buchanan | Illinois. |
| Charles P. S. Beerend | Wisconsin. |
| Frank H. Stafford | Chicago. |
| Watson Martin | Chicago. |

HONARA DEGREE.

| | |
|---|---|
| Daniel B. Freeman | Chicago. |
| Charles W. Stutenroth | South Dakota. |

### Ohio College of Dental Surgery.

The forty-fifth annual commencement of the Ohio College of Dental Surgery, was held at the Scottish Rite Cathedral, Cincinnati, Ohio, on Wednesday, March 11th, 1891, at 8 o'clock, P. M.

The number of matriculates for the session was two hundred and ten.

The degree of D.D.S. was conferred on the following persons by D. W. Clancey, M.D., D.D S. :

| NAME. | RESIDENCE. |
|---|---|
| Emanuel Joe Abeles | Ohio. |
| Benjamin Erskine Ashby | Kentucky. |
| Travers Barrett | Missouri. |
| Andrew Hunter Boyd | Tennessee. |
| William Aristades Burnett | Ohio. |
| John Wesley Buzzerd | Ohio. |
| Charles Harrison Campbell | Ohio. |
| Clinton Emmett Case | Indiana. |
| Jerome Bonaparte Chaffee | Missouri. |
| Frank Riley Chapman | Ohio. |
| John Marcellas Chase | Ohio. |
| Mrs. Fannie Cooper. | Kentucky. |
| Harlan Breckenridge Copsey | Calif'nia |
| Donald Dean Cornell | Iowa. |
| Ben Brodie Cory | California. |
| Will McAnney Coryell | Indiana. |
| Edwin Pierce Cunningham, Jr. | Ohio. |
| Hugh C. Davidson | Ohio. |
| Gilbert Wesley DeCamp, Jr. | Ohio. |
| Will Herbert Ervin | Indiana. |
| Frank Eugene Faveret | Ohio. |
| Jay Carpenter Foulk | Ohio. |
| Thomas Webster Freeman | Canada. |
| Leander B. Furman | Pennsylvania. |
| Charles Herschel Geiger | Ohio. |
| Joseph William Gercken | Ohio. |
| Harry Stewart Gilson | Pennsylvania. |
| Edward Crum Grant | Ohio. |
| John Henry Hines | Ohio. |
| Allen Howe | Minnesota. |
| Charles Woodward Hubbell | Ohio. |
| Elmer Ellsworth Israel | Indiana. |
| James Warren Jackson | Ohio. |
| Harry Joseph Johnson | Ohio. |
| Lester Hicks Knapp | New York. |
| Albert Amos Kulmer | Ohio. |
| Clayton Vincent Lanum | Ohio. |
| William Sherman Leeds | Indiana. |
| William Stanton Locke | Ohio. |
| John Floyd Lockhart, M.D | Kentucky. |
| Lewis Alfred Long | Pennsylvania. |
| Cornelius Vanderbilt Mallory | Ohio. |
| John Watson McAbee | California. |
| Frank M. McCarty | Indiana. |
| W. Owen McGaughy | Mississippi. |
| Edward Alexander Mehaffey | Texas. |
| Edwin Christopher Meyer | Indiana. |
| George Murray Miller | Canada. |
| Joseph Vincent Miller | Wisconsin. |
| Elmer Carlisle Moore | Ohio. |
| William Newton Morgan | Ohio. |
| John Franklin Outcalt | Ohio. |
| Clark Stephenson Pearce | Illinois. |
| Frank A. Pfouts | Ohio. |
| Joseph Rockwell Price | Massachusetts. |
| William John Reid | Ohio. |
| Charles Dale Richey | Pennsylvania. |
| Robert Paul Scudder | Ohio. |
| George Marcellus Shafer, M.D | Ohio. |
| Charles L. Slutter | Ohio. |
| Herold Ellsworth Smith | Ohio. |
| Stephen Clyde Smith | Indiana. |
| A. Cristie Smysor | Ohio. |
| William Daniel Snyder | Ohio. |
| Frank McGee Sparks | Indiana. |
| John Anson Bering Srofe | Ohio. |
| Edward Russell Stevenson | Ohio. |
| William W. Tarr | Ohio. |
| Benjamin Cackayne Taylor | Ohio. |
| Herman B. Van Tres | Ohio. |
| Henry Emanuel Weick | Ohio. |
| Albert Weiler | Ohio. |
| John Fred. Werner, Jr | Michigan. |
| Charles S. Williams | Ohio. |
| Eugene Frank Winchet | Ohio. |

Total, 75.

### New York College of Dentistry.

The twenty-fifth annual commencement of the New York College of Dentistry, was held in Chickering Hall, New York City, Tuesday evening, March 10th, 1891.

The number of matriculates was two hundred and eighty-two. The degree of D.D.S. was conferred on the following persons by Hon. J. Hampden Robb, President of the Board of Trustees:

Adair, Frank Lincoln.

Adler, Gustav Herman.

Abbanesius, Otto Henry.

Aldred, Oliver Harvy.

Allen, Charles Edward.

Amyot, Bruno Edmund.

Argilagos, Alfred Augustin.

Baker, David Coddington.

Kadelbach, Albert R.

Knapp, James Franklin.

Knight, George Winthrop.

Leverich, Charles Bornman.

Lockwood, Frank Granger.

Luckey, Charles Molton.

Ludlum, Fred. Willett.

McCormic, Samuel George.

Barrett, George Francis.

Bassett, Charles Gibson.

Biava, John.

Bromberg, Bernard Benedict.

Broughton, John Glover.

Brown, Mark Hart.

Bullis, Steward Franklin.

Burgess, Fred. Lane.

Byrne, David Walter.

Bird, Joseph Modesto.

Bradfield, Thomas Naylor, Jr.

Cannon, Charles Mousby.

Chauvet, Joseph Edward.

Clayton, Walter Hassell.

Cleveland, Edward Thomas.

Collett, Frederick George.

Dickson, James Lenny.

Douglas, William Henry, M.D.

Downs, Samuel John.

Emerson, Charles Howard.

Fielding, Frank Arthur.

Fones, Alfred Civilion.

Garside, William Owen.

Gilson, Clarence Delbert.

Graves, Henry Whitmill.

Guilshan, Henry William.

Hanks, John Tomlinson.

Hannemann, F. Theo. A.D., M.D.

Hardy, James Walter.

Hart, Morris Philip.

Hess, Louis.

Hornug, William Augustus.

Hough, William Stewart.

Jacobson, Benjamin.

McNerny, Frederick.

Meeker, John Burton.

Merwin, Robert Eugene.

Messerschmitt, Frederick.

Minor, Waldo Henry.

Morhard, Frank Louis.

Morris, Frank.

Moss, Alexander Henry.

Newhall, Channing Angelo.

Nicholas, Henry Skidmore.

Norton, Samuel John Leake.

Ochoa, Raul.

O'Neill, William Robert.

Overton, Mordecal Horace.

Park, William Brush.

Payne, Winthrop Bryant.

Perry, Andrew Jackson.

Pfeiffer, Henry Peter.

Phillips, Frank Leonard.

Pomeroy, Irving Herbert.

Pravost, William Douglas.

Potter, Louis Edgar.

Quinlan, Joseph Stanislaus.

Reeves, George Noakes.

Rice, Walter Delavan.

Roche, Thomas Henry, B. S.

Root, Charles William.

Root, John Calvin.

Russell, Samuel Phillips.

Snedaker, John Frederick.

Stuki, August George.

Van Geison, Walter Francis.

Vernon, Louis Elvin.

Weinlandt, Francis.

Willis, John Frederick.

### Vanderbilt University—Department of Dentistry.

The twelfth annual commencement exercises of the Department of Dentistry of Vanderbilt University was held at Union Chapel, Nashville, Tennessee, March 25th, 1891.

The number of matriculates for the session was one hundred and thirty-five.

The degree of D.D.S. was conferred by W. F. Tillett, Vice-Chancellor, on the following persons:

| NAME. | RESIDENCE. |
|---|---|
| R. L. Allen | Alabama. |
| J. D. Adair | Alabama. |
| Archie Boales | Kentucky. |
| R. H. Burks | Kentucky. |
| David Combs | Texas. |
| Wm. Chapman | Illinois. |
| Arthur Corbin | Michigan. |
| J. E. Combs | California. |
| J. G. Cutliff | Georgia. |
| W. W. Corby | Alabama. |
| B. S. Davis | Mississippi. |
| B. N. Durpee | Alabama. |
| J. A. Dale | Indiana. |
| O. C. Delhommer | Louisiana. |
| R. D. Griffis | Texas. |
| Manuel Garfias | Mexico. |
| J. M, Graham | Tennessee. |
| G. H. Hudson | Alabama. |
| J. H. Hatcher | Missouri. |
| O. P. Hope | Missouri. |
| L. T. Hallum | Tennessee. |
| E. F. Hickman | Tennessee. |
| N. R. Holcomb | North Carolina. |
| Hamet Jordan | Virginia. |
| J. D. Killian | Alabama. |
| R. J. Mills | Kentucky. |
| S. J. Martin | Kentucky. |
| E. F. Morris | Texas. |
| J. W. Perkins | Alabama. |
| G. S. Pearcy | Tennessee. |
| W. S. Parker | Mississippi. |
| C. W. Patterson | Mississippi. |
| R. A, Rush | Mississippi. |
| J. D. Smith | Tennessee. |
| J. D. Stephens | Alabama. |
| W. E. Swind | Missouri. |
| B. F Storne | South Carolina. |
| W. L. Smith | New York. |
| Thos. Towles | Kentucky. |
| J. W. Thomas | Kentucky. |
| J. S. Ward | Tennessee. |
| J. P. Williams | Georgia. |
| G. J. Williams | Texas. |

### Philadelphia Dental College.

The twenty-eighth annual commencement of the Philadelphia Dental College, was held at the Academy of Music Thursday, February 26th, 1891, at 8 P. M.

The number matriculates for the session was three hundred and fifteen.

The degree of D.D.S. was conferred on the following persons by Chas. P. Turner, M. D.

| NAME. | RESIDENCE. |
|---|---|
| Aaron, Joseph B | New York. |
| Agnew, Thos. H | Canada. |
| Albee, Edmund H | New Hampshire. |
| Aldred, H. Augustus | Pennsylvania. |
| Andler, George J | Massachusetts. |
| Archer, Cuthbert C | West Indies. |
| Baker, Albert R | Canada. |
| Barrett, Edward C | Maine. |
| Beach, Louis L | Connecticut. |
| Black, Fred L. | New Brunswick. |
| Blanchard, J. E., Jr. | Louisiana. |
| Booth, Frank E | Kentucky. |
| Booth, William H | Pennsylvania. |
| Bolger, William M | Pennsylvania. |
| Bragdon, Charles S. | Maine. |
| Brown, Clark W | Pennsylvania. |
| Burdates, Oscar W. | West Virginia. |
| Buss, John G., L. D. S | France. |
| Caldwell, Wm. S., Jr | Pennsylvania. |
| Callaway, William L | Missouri. |
| Capwell, Courtland G | Rhode Island. |
| Carman, Thomas D | New York. |
| Caspersonn, Edward | Australia. |
| Chatham, Edward R | New York. |
| Chase, Arthur L | Maine. |
| Chapman, B. O | New York. |
| Chapman, Samuel W. | Connecticut. |
| Cohn, Julius | Roumania. |
| Cook, William S | Pennsylvania. |
| Comins, A. Olin | Connecticut. |
| Corkhill, Martha C. | Pennsylvania. |
| Crawford, Elmer | Pennsylvania. |
| Damon, William A. | Connecticut. |
| Davey, Reuben S. | New York. |
| De Garis, Arthur | New York. |
| De Les Derniers, W. R | Canada. |
| Dennis, N. P., M. D | California. |
| Dienst, Alexander | Missouri. |
| Dungan, G. A | California. |
| Eisenbrand, Geo. F | Minnesota. |
| Elder, Jacob S | Pennsylvania. |
| Elliott, J. T | Kentucky. |
| Engel, William S. | Pennsylvania. |
| Fickes, William L | Ohio. |
| Fry, H. M | Pennsylvania. |
| Fyffe, Charles, Jr | Australia. |
| Gelston, William H | New Jersey. |
| Graves, James L | Oregon. |
| Griswold, Monroe | Connecticut. |
| Halsey, W. Edward | New York. |
| Halstead, William T | Australia. |
| Hambley, Joseph T | New Jersey. |
| Hastings, Wm. A | New York. |
| Havice, Charles T | Pennsylvania. |
| Haynes, Harry W | Maine. |
| Henderson, Geo. A | Pennsylvania. |
| Hewish, Herold C. | Canada. |
| Hill, Ambrose L | New York. |
| Hinder, Arthur G. A | Australia. |
| Hodgkins, Harvey L | Maine. |
| Hoopes, Charles W | Pennsylvania. |
| Hohl, Carl F. | Indiana. |
| Hunter D. Elmer | Canada. |
| Ichinoi, Masatsune | Japan. |
| Jackson, J. Holmes | Canada. |
| Jessop, William D | Pennsylvania. |
| Johnston, David W | New Jersey. |
| Justus, Peter | Portugal. |
| Kalbfus, Joseph | Pennsylvania. |
| Kerr, Donald E | Canada. |
| King, Jesse D. | Missouri. |
| King, Thomas J | Maine. |
| Lewin, Walter W. | England. |
| Lewis, William D | New York. |
| Lindstrom, C. Richard | Sweden. |
| Lofland, Howard | Delaware. |
| Logan, William C | Oregon. |
| Losee, Irving H | New York. |
| MacMullen, David A | California. |
| Maley, Nixon | Canada. |
| Martin, Emile A | South America. |
| Martin, Benjamin B | Pennsylvania. |
| Maschke, George | Ohio. |
| McCullough Piercy M.B. | Pennsylvania. |
| McDowell, Will | Indiana. |
| McHugh, John, Jr | New York. |
| McNeille, Charles S | New York. |
| Miller, Frank P | New York. |
| Nakamura, Kazuyos | Japan. |
| Nadel, Sigmund | Austra. |
| Neel, Ralph A | Pennsylvania. |
| Nickerson, Geo. Q | Maine. |
| Noling, Isaac O | Illinois. |
| Nones, Henry B | Pennsylvania. |
| O'Brien, John W | New York. |
| Parker, Fred H | Nova Scotia. |
| Parkhurst, Fenimore | New York. |
| Percival, Winston G | Pennsylvania. |
| Pratt, Walter I. | Utah. |
| Preston, Nathaniel E | Massachusetts. |
| Pressey, Burt | New Jersey. |
| Quattlebaum, E. G | South Carolina. |

| NAME. | RESIDENCE. | NAME. | RESIDENCE. |
|---|---|---|---|
| Randles, Richard N | Canada. | Stockton, Henry E | North Carolina. |
| Reid, David P | New York. | Sullivan, Joseph C | Alabama. |
| Rhoads, Frank O | Missouri. | Sweeney, John | Pennsylvania. |
| Richardson, E. L | Maine. | Taube, Friedrich Wm | Germany. |
| Robb, John W | Canada. | Tener, Robert W | West Virginia. |
| Roberts, Fred A | New Brunswick. | Thistlewood, B. R | Illinois. |
| Robson, John L | Pennsylvania. | Thomas, Jenkyn, Jr | Utah. |
| Roe, C. Harry | Pennsylvania. | Tompkins, Henry H | New York. |
| Root, Frederick W | Massachusetts. | Traver, Frank A | Wisconsin. |
| Ryan, James M | Massachusetts. | VanAllen, W. J. Frank | Canada. |
| Shirlow, Wesley W | Australia. | Velescu, Leo | Roumania. |
| Shillinger, Eugene A | New York. | Waite, Ralph B | New York. |
| Slegel, Walter R | Pennsylvania. | Warner, Frank W | New York. |
| Smith, Clarence U | New York. | Wells, Claude E | Missouri. |
| Snider, William C | New York. | Wells, John I | Missouri. |
| Solomons, Robert M | South Carolina. | Wheeler, Gilman A | Vermont. |
| Spangler, Geo. M | Pennsylvania. | Whitcomb, Frank E | Maine. |
| Sprout, William A | Pennsylvania. | Winterfield, Morris P | New York. |
| Stackhouse, J. A | Canada. | Wright, William E | Delaware |
| Stamm, Carl P | Pennsylvania. | Yocum, William L | Pennsylvania. |
| Stark, Nathan P | Pennsylvania. | Zellers, R. E | Texas. |
| Steele, N. D | Canada. | Ziegler, Charles L | California. |

Graduates, 146.

### Dental Department of the Northwestern University.

The commencement exercises of the University Dental College, Department of the Northwestern University, were held in connection with the Medical Department—Chicago Medical College—at Central Music Hall on April 28th at 2:30 P. M.

The degrees were conferred upon the graduating classes by President Henry Wade Rogers L. L. D. and the Doctorate address was delivered by Proffessor N. S. Davis Sr., Dean of the Medical Faculty:

The following is the list of the Dental graduates.

| NAME. | RESIDENCE. | NAME. | RESIDENCE. |
|---|---|---|---|
| Ellsworth Goldthorp | Iowa. | Alexander Clarence Murchison | Ills. |
| William Edward Harper | Illinois. | William B. Winget | Illinois. |

### The Columbian University, Dental Department, Washington, D. C.

The Columbian University, Dental Department, of Washington, D. C., held its fourth annual commencement at Lincoln Music Hall, Washington, D. C., on Thursday, March 19th, 1891, at 8 P. M.

The number of matriculates for the session was nineteen.

The degree of D.D.S. was conferred on the following persons by Dr. J. C. Welling, M.D., D.D.S:

| NAME. | RESIDENCE. | NAME. | RESIDENCE. |
|---|---|---|---|
| Jonathan R. Hagan | Virginia. | Benjamin F. Odell | Illinois. |

### Pennsylvania College of Dental Surgery.

The thirty-fifth annual commencement of the Pennsylvania College of Dental Surgery was held at the Academy of Music, Philadelphia, Pennsylvania, Friday evening, February 27th, 1891, at 8 o'clock.

The number of matriculates for the session was two hundred and fifty-one.

The degree of D.D.S. was conferred on the following persons by I. Minnis Hayes, President of the College:

| NAME. | RESIDENCE. |
|---|---|
| Wm. Alexander | Pennsylvania. |
| E. W. Armistead | Virginia. |
| Chauncey Bachman | New York. |
| N. J. Baker | New York. |
| H. P. Baldwin | New Hampshire. |
| Reberto Barrera | U. S. Colombia. |
| T. W. Bortree, M. D. | Pennsylvania. |
| Richard Y. Bates | Minnesota. |
| L. B. Bowie | Pennsylvania. |
| Chas. G. Bowles | Michigan. |
| Wm. P. Brown | Minnesota. |
| Chas. M. Brooks | New York. |
| J. A. Brunet | Chili. |
| Melquiades Bruges | U. S. Colombia. |
| J. P. Calvert | Pennsylvania. |
| George Campusano | Pennsylvania. |
| C. W. Chapman | California. |
| Cunningham Clark | Pennsylvania. |
| Edw. Conover | New Jersey. |
| Sam. M. Cooley | Indiana. |
| A. G. Courtney | New York. |
| A. E. Cribbs | Pennsylvania. |
| Z. H. Curry | Pennsylvania. |
| T. J. Crymes | South Carolina. |
| C. T. Dahlin | Illinois. |
| H. H. Donaldson | Pennsylvania. |
| Geo, W. Dunbar, Jr. | New York. |
| C. B. Edmiston | Pennsylvania. |
| Max J. Fischel | Pennsylvania. |
| S. W. Frazier | Ohio. |
| J. W. Fulstone | Nevada. |
| G. L. Grier | Delaware. |
| M. E. Grossman | Honolulu, H. I. |
| G. W. Gunther | Germany. |
| E. T. Grosvenor | Michigan. |
| J. G. St. Hammond | Sweden. |
| W. J. Hardie, L.D.S. | Scotland. |
| W. G. Hayes | Pennsylvania. |
| J. J. Heffernan | Pennsylvania. |
| L. W. Heinlein | Ohio. |
| Geo. H. Heist | Virginia. |
| H. M. Hertig | Pennsylvania. |
| Windfield M. Hubler | Pennsylvania. |
| Morgan L. Hulme | Pennsylvania. |
| E. L. Irving | Minnesota. |
| F. W. Ivory | Canada. |
| G. R. Johnson | Canada. |
| H. S. Keepers | Pennsylvania. |
| W. S. Kelly | Pennsylvania. |
| E. S. Kirkpatrick | Pennsylvania. |
| Chas. G. Koester | New York. |
| Louis F. Koehler | Pennsylvania. |
| Ricardo Larenas | Chili. |
| H. W. Leightner | Pennsylvania. |
| A. M. Lewis | Minnesota. |
| Wm. E. Linn | Ohio. |
| B. M. Loar | Pennsylvania. |
| F. A. Lackner | Canada. |
| Wm. J. Longnecker | New York. |
| Mateo Lucena | Venezuela. |
| Felice Maddalena | Italy. |
| A. P. Matson | Connecticut. |
| A. B. Miller | Pennsylvania. |
| Morton Mills | New Jersey. |
| Fred. Miltenberger | Germany. |
| C. O. Morris | Pennsylvania. |
| E. C. Musser | Pennsylvania. |
| S. J. MacMains | Pennsylvania. |
| Ellen MacMurray | Pennsylvania. |
| H. McIntyre | Canada. |
| A. L. Parker | New Hampshire. |
| V. A. Pazmino | U. S. Colombia. |
| Henry A. Phillips | England. |
| E. E. Phipps | Pennsylvania. |
| Wm. C. Porter | England. |
| Jaquin Preito | U. S. Colombia. |
| C. G. Richardson | Penn-ylvania. |
| F. W. Rice, M. D. | Pennsylvania. |
| Louis S. Robenshon, M.D. | Russia. |
| Emetrio Serrand | U. S. Colombia. |
| Jules Simond | Switzerland. |
| Geo. N. Slater | Pennsylvania. |
| C. P. Shoemaker | Pennsylvania. |
| F. W. Steinbock | Pennsylvania. |
| H. J. Stewart | New York. |
| T. B. Stewart | Pennsylvania. |
| Ellwood Tate | Pennsylvania. |
| W. K. Thorp | Pennsylvania. |
| C. L. True | New Hampshire. |
| Chas. S. Voorhis | Pennsylvania. |
| S. D. Weber | Pennsylvania. |
| W. R. Wilkinson | Canada. |
| Emily W. Wyth, M.D. | Pennsylvania. |
| Emma C. Wygant | New York. |

### University of Iowa—Dental Department.

The ninth annual commencement of the Dental Department of the State University of Iowa took place at the Opera House, Iowa City, Iowa, on Monday evening, March 9th, 1891.

The annual address was delivered by William Stevens Perry, D.D., L.L.D.

The number of matriculates for the session was one hundred and sixty-one.

The degree of D.D.S. was conferred on the following persons by Charles A. Schaeffer, Ph. D., President of the University.

| NAME. | RESIDENCE. | NAME. | RESIDENCE. |
|---|---|---|---|
| R. C. Amrine | Illinois. | Thomas Gormley | Mt. Vernon. |
| J. O. Applebee, A. M | Red Oak. | C. L. Girls | Muscatine. |
| H. O. Allen | Wapello. | James Galway | Wisconsin. |
| C. F. Adams | Independence. | J. E. Hawthorne | Illinois. |
| D. C. Brett | Morrison. | L. C. Hall | Burlington. |
| G. H. Barker | Massachusetts. | P. R. James | Belmond. |
| G. H. Belding | Calmar. | C. O. Jerrell | Mt. Pleasant. |
| E. Bumgardner, B. S | Kansas. | L. E. Kaltenbach | Wisconsin. |
| J. W. Billings | Union. | C. E. Laird | Newton. |
| A. L. Brown | Perry. | J. F. Leigh | Dyersville. |
| D. J. Brown | Waterloo. | Cora G. Little | Perry. |
| Frank Ball | Nebraska. | W. D. McErlain | Dyersville. |
| N. Burdick | Davenport. | Jas. McNeil | Mason City. |
| C. D. Bemiss | Washington. | W. W. Moorehead | Illinois. |
| Frank Bickel | Illinois. | G. C. Money | Beaman. |
| R. S. Bandy | Fairfield. | J. B. Moats | Illinois. |
| W. J. Coughlan | Colfax. | C. A. Marshall | Nebraska. |
| A. L. Currie | Earlville. | A. E. Osborn, C. S | Summer. |
| George Cogley | Clarinda. | J. L. Riggs | Castalia. |
| V. K. Chandler | Perry. | J. M. Raugh | Maxwell. |
| G. E. Chambers | Wisconsin. | S. B. Reque | Minnesota. |
| W. C. Davis, B. S | Oxford. | M. A. Robinson | Maquoketa. |
| J. W. Downey, M. D | State Center. | W. F. Ryburn | Illinois. |
| S. G. Dowdey | Cherokee. | H. C. Reeves | New York. |
| J. B. Ellis | Maquoketa. | C. C. Smith | Illinois. |
| W. E. Fish | Kellogg. | L. F. Steuerwald | South Dakota. |
| C. A. Fuller | New York. | M. L. Spencer | Waterloo. |
| L. L. Foote | Minnesota. | W. C. Schoemaker | Muscatine. |
| L. S. Forbes | Fayette. | J. P. Von Lackum | Waterloo. |

The reports from other colleges will appear in the July REGISTER.—[EDITOR.

THE *Medical Record* reports that Dr. M. Sternberg has found something new in human osteology. It is only a little hole in the sphenoid bone, which he calls the "canalis cranio-pharyngeus." It is best seen in the skull of a child four years of age.

